# Does “Individual Placement and Support” Satisfy the Users’ Needs?

**DOI:** 10.3389/fpubh.2015.00160

**Published:** 2015-06-16

**Authors:** Sandra Viering, Matthias Jäger, Carlos Nordt, Franziska Bühler, Bettina Bärtsch, Hansjörg Leimer, Peter Sommerfeld, Wulf Rössler, Wolfram Kawohl

**Affiliations:** ^1^Department of Psychiatry, Psychotherapy and Psychosomatics, Centre for Social Psychiatry, University Hospital for Psychiatry Zurich, Zurich, Switzerland; ^2^Institute for Social Work and Health, School of Social Work and Health, Olten, Switzerland; ^3^Laboratory of Neuroscience, LIM 27, Institute of Psychiatry, University of São Paulo, São Paulo, Brazil; ^4^University of Zurich, Zurich, Switzerland

**Keywords:** supported employment, individual placement and support, satisfaction, rehabilitation, mental illness

## Abstract

This study aims to investigate clients’ satisfaction with individual placement and support (IPS) at the University Hospital for Psychiatry Zurich (PUK). Furthermore, this study aims to investigate if clients feel the approach of IPS as a useful approach to fulfill their needs. One hundred twenty-five people were recruited from one of the three IPS services of PUK and were asked to complete a structured questionnaire. The following IPS services were available: (i) randomized controlled trial (RCT) ZHEPP (www.zhepp.ch), (ii) RCT ZInEP (www.zinep.ch), and (iii) us clinical supported employment service of PUK (IPS-PUK). The clients mostly indicated that IPS was generally useful and fitted their needs. Overall satisfaction of the participants with the IPS services of the PUK was very high. Furthermore, client satisfaction and symptom severity are inversely associated. In conclusion, participants of the IPS services received the support they were looking for. This means that the approach of IPS fits the needs of different patient groups and can be used without any modifications. The most important limitation is the unequal group sizes. Therefore, the obtained results need to be strengthened by future research.

## Introduction

In the last 20 years, research on vocational rehabilitation revealed that supported employment (SE) according to the principle “first place, then train,” produces better competitive employment outcomes compared to classical vocational rehabilitation based on the “first train, then place” approach (1). In addition, earlier studies demonstrated that SE leads to better quality of life, less psychopathology, and lower drop-out rates (2).

In 1993, Becker and Drake (3) defined the individual placement and support (IPS) model, a variant of SE that is based on eight principles: (a) placement in competitive employment market, (b) focus on client preferences, (c) individualized support, (d) close cooperation with the care system, (e) openness to anyone who wants to work in the competitive employment market, (f) rapid job search, (g) job development, and (h) network of potential employers built up by the job coaches. Additionally, the clients are free to choose the number of appointments with their job coach.

Until now, almost all studies on IPS focused primarily on the question if IPS produces better employment outcomes than traditional vocational rehabilitation (TVR) or sheltered workplaces in general. Next to that, research focused on the quality of life and the psychopathology of clients using IPS and TVR, while little research has been done on clients’ satisfaction. It has been shown that satisfaction with IPS services is linked to better employment outcome and leads to higher motivation among the clients (4). However, since research regarding patient satisfaction with treatments in general is scarce (5) further studies are needed. The research should consider the question whether IPS fits different needs of different groups (i.e., persons with mental illnesses who are unemployed and want to work in the competitive employment market or employees with mental illnesses who have problems with their current job situation).

The services of the University Hospital of Psychiatry Zurich (PUK) include Supported Employment, which was used as a reference service. Moreover, PUK participated in an international randomized controlled trial (RCT) called enhancing the quality of life and independence of persons disabled by severe mental illness through supported employment life (EQOLISE) (6), which focused mainly on the effect of IPS on people with mental disorders concerning various aspects such as salary, job tenure, and quality of life.

The study presented here investigates the satisfaction of the IPS services users (IPS-PUK) and of the participants of two RCT conducted at PUK: SE trial of the Zurich Program for Sustainable Development of Mental health services (ZInEP) and the Zurich Reintegration-Pilot-Project (ZhEPP).

This article aims to clarify the following questions:

(1) Do IPS services satisfy users’ needs?
(2) How is the satisfaction with the clinical IPS services in general?
(3) Is there an association of satisfaction with psychopathology?

## Materials and Methods

### Design/setting

The data were collected using a structured questionnaire developed specially for the purpose of the study. The participants of the study were recruited from one of three different IPS services available at PUK. If a client refused to participate in the study no record was made. Before participants answered the questionnaire, they were informed about purpose and procedure of the study. Afterwards, they were asked to sign an informed consent. Participation was voluntary and anonymous. The questionnaire was handed out by the job coaches and completed at the beginning of the coaching session requiring about 10–15 min. The participants received no remuneration.

### Participants

The participants were recruited from three different IPS services available at PUK: (i) RCT ZHEPP^1^ (7) RCT ZInEP^2^ (8) and clinical SE service of PUK (IPS-PUK). In total, 125 individuals participated (ZhePP = 53, SE = 59; ZinEP = 13). The sample consisted of 55 (44%) males and 70 (56%) females, with a mean age of 43.22 years (SD = 10.78). The three groups did not differ significantly regarding age and gender of the participants.

Inclusion criteria for the current study were a 18 years of age or older, a diagnosed mental disorder and participant’s goal to work in competitive employment market. Furthermore, participants had to be engaged in one of the IPS services of PUK at the time of study. Exclusion criteria were severe organic disorder (ICD 10: F0) or mental retardation (ICD 10: F7). Nevertheless, the participants’ characteristics differed slightly between the groups. ZHEPP participants were recruited with support form IV-office Zurich. Both employed and unemployed individuals were allowed to participate. However, most of the participants included in this study were unemployed. ZInEP were recruited from six outpatient clinics in the Canton of Zurich. Individuals included in the study had to be unemployed for at least 3 months. Users of the clinical SE service of PUK (IPS-PUK) enrolled themselves in the program. Most participants in this group enrolled because they still had a job but needed help in daily work. At the beginning of data collection, all participants were engaged in one of the three IPS services. However, some participants were in one group (e.g., IPS-PUK) at the beginning of the study and changed to another one (e.g., ZhEPP) later. The current employment status of the participants is not known. However, different group characteristics allowed to assume the employment status based on the group membership. All groups received similar IPS services and all services were located at PUK.

### Measures

Client satisfaction with IPS was evaluated using a questionnaire based on the ZüPaZ (“Zürcher Fragebogen zur Patientenzufriedenheit”; i.e., Zurich questionnaire of patients’ satisfaction) (9) and the ZUF-8 (“Zufriedenheitsfragebogen”; i.e., Satisfaction questionnaire) (10). We chose eight items from the ZüPaZ and modified them. Those modifications included wording adaptation to fit the SE terminology (e.g., “Job Coaches” instead of “doctors”; “Coaching goals” instead of “Treatment goals”). The entire ZUF-8 was included without any modifications. Both scales include items rated on Likert scale. The score ranged from 1 to 4 with higher values indicating higher satisfaction. Based on these two questionnaires, the overall satisfaction score of the sample was conducted. Additionally, a modified version of the “Lebensführungssystem” (Life-leading system) (11) was included. The main goal of this questionnaire was to acquire knowledge, about the individual usefulness of IPS for each client. The questions included 11 dichotomous items, some of them not directly related to IPS (e.g., to deal with addictive drugs). These items were chosen to discriminate between IPS and other tasks. The clients were asked to select the three items from which they profit the most.

The symptomatology of the clients over the past 4 weeks was assessed with the German version of the Symptom Check List SCL-10 (12), a short version of the SCL-90. Questions regarded symptoms of interpersonal sensitivity, depression, anxiety, phobic anxiety, and psychoticism. The scale ranged from 0 (“no suffer”) to 4 (“suffered very strong”).

### Statistics

Statistical analysis was conducted using SPSS 20.0. Descriptive analysis regarding sex, age, group membership, and duration of participation in one of the IPS services was computed.

To test for normal distribution of continuous variables the Kolmogorov–Smirnov test was used. As the variables were not normally distributed, Kruskal–Wallis test was performed to test for group differences of the continuous variables. Crosstabs were implemented in order to investigate whether the categorical variables, in particular, variables from Life-leading system, differed between the groups.

Descriptive analysis was conducted regarding the overall satisfaction level with the IPS offer and the overall symptomatology of the clients. Spearmen correlations were performed between satisfaction measures and symptomatology.

## Results

The mean participation duration in any of the SE services was *M* = 1.82 (SD = 1.23) years. Participants from ZInEP took part the longest (*M* = 2.38, SD = 0.87), followed by IPS-PUK (*M* = 1.92, SD = 1.49) and ZhEPP (*M* = 1.58, SD = 0.89).

The results for the life-leading system-questionnaire are depicted in Table 1. A significant association between group membership and the subjective importance of the item “finding a job” was found. Most subjects who rated this item as important were participants of ZInEP. However, ZhEPP and IPS-PUK also rated this item as important. Further group differences regarding the item “IPS gave me good support while I was working” were found. While the majority of the IPS-PUK sample considered this item as important, only about the half of the ZhEPP sample and just few of the ZInEP subjects considered the support by IPS important for daily work. Group differences were also found regarding the items “IPS helps me to deal with my boss,” “IPS helps me to deal with spare time,” and “IPS helps me to gain more self-esteem.”

**Table 1:** **Group differences in individual usefulness of IPS**.

IPS supports me…	IPS-PUK rated item as important (%)	ZhEPP rated item as important (%)	ZInEP rated item as important (%)	*p*	χ^2^	df
…to find a job	47	48	84	0.039*	6.49	2
…while I was working	70	36	15	<0.001*	19.75	2
…to deal with my colleagues	23	9	23	1.472	3.84	2
…to deal with my boss	39	25	0	0.015*	8.45	2
…to deal with my social life	14	26	23	0.264	2.667	2
…to deal with my doctor and carer	32	21	15	0.294	2.45	2
…to deal with my spare time	4	21	0	0.006*	10.36	2
…to organize my daily structure	12	25	23	0.235	2.89	2
…to gain more self-esteem	37	60	69	0.017*	8.15	2
…to build more capacities	23	32	31	0.535	1.251	2
…to deal with addictive drugs	0	0	0	–	–	–

*p > 0.05.

*IPS, individual placement and support; IPS-PUK, IPS-standard offer of PUK; ZhEPP, Zurcher Reintegration Pilot Project; ZInEP, Zurich program for Sustainable Development of Mental Health services*.

Data based on the “life-leading system.”

The Kolmogorov–Smirnov test showed that symptomatology [*D*(125) = 0.90, *p* = 0.014] and satisfaction [*D*(125) = 0.15, *p* < 0.001] were not normally distributed. Overall symptomatology of the whole sample was low (*M* = 1.18, min = 0.00, max = 3.78, SD = 0.77). Group comparison indicated that there was no significant difference in overall symptomatology between groups [*H*(2) = 4.70, *p* = 0.095, IPS-PUK = 57.89, ZhEPP *M* = 71.04, ZInEP *M* = 53.42]. Overall satisfaction with the IPS offer was high (*M* = 3.57, min = 1.57, max = 4.00, SD = 0.35). Furthermore, there were no significant differences in overall satisfaction between the groups [*H*(2) = 0.11, *p* = 0.95, IPS-PUK *M* = 63.69, ZhEPP *M* = 62.98, ZInEP *M* = 59.96]. Higher satisfaction with IPS services was associated with lower symptomatology (*r* = −0.278, *p* = 0.002).

## Discussion

Our study sample includes one group with most participants already having a job (IPS-PUK) and two groups in which most persons were looking for a job (ZInEP; ZhEPP). IPS-PUK participants rated the items of the Life-leading system that are associated with daily work as more important than the ZhEPP and ZInEP participants. ZInEP participants scored the item “IPS supports me to find a job” especially high. This answers our first research question, if IPS services satisfy the users’ needs. Participants of IPS services, who were mostly seeking a job (ZInEP), got what they were looking for, as did those participants who needed support in their daily work (IPS-PUK). Hence, the approach of IPS demonstrates its flexibility to fulfill the needs of two different groups. This study shows that there is no difference concerning satisfaction between the groups, as all participants were highly satisfied with IPS. Although in most satisfaction studies in health care system individuals are generally highly satisfied (13), our study showed that it is important to consider in detail, what makes people satisfied with vocational rehabilitation services (14). These details should be investigated more thoroughly in the future.

As satisfaction correlates with motivation (15) and motivation correlates with coaching success (16), the knowledge of clients’ satisfaction is important. This knowledge will help to fill possible gaps in the IPS services.

This study showed that participants with fewer psychiatric symptoms were more satisfied with IPS. However, the symptomatology of the entire sample was low. Therefore, an interesting question arises: does IPS lead to fewer psychiatric symptoms or do IPS participants show fewer psychiatric symptoms to begin with? Further research should undoubtedly address this issue. Becker et al. (17) found poor coping with symptoms as an influential factor for job satisfaction. Therefore, we expect the symptomatology to have a substantial influence on satisfaction with IPS. Indeed, this association was observed in the present study. Participants with fewer symptoms were more satisfied with the IPS offer.

Nevertheless, limitations of this study should be considered. First, most participants in the IPS-PUK group had a job, whereas most participants in ZInEP and ZhEPP were looking for a job. Therefore, the group differences probably would be higher if the groups had clear inclusion criteria regarding the job status.

Moreover, a limitation is the unequal distributions of the groups. The total sample size is sufficiently large, but the groups have unequal sizes, i.e., ZInEP contains 13 participants and ZhEPP and SE more than 50 participants each.

Previous studies show that the characteristics of the job coaches are very important to the success of job coaching (18). Some items of Life-leading system contained satisfaction with the treatment by the job coach. In our study, the satisfaction was linked to the treatment by the job coach. Some job coaches supervised participants several groups (e.g., ZhEPP and IPS-PUK). This could influence the results of group differences in satisfaction.

Furthermore, the satisfaction results might have been influenced by the job coaches handing out the questionnaire and being present while clients answered the questions.

## Conflict of Interest Statement

The authors declare that the research was conducted in the absence of any commercial or financial relationships that could be construed as a potential conflict of interest.

## Footnotes

^1^www.zhepp.ch

^2^www.zinep.ch

## Abbreviations

EQOLISE, severe mental illness through supported employment life; IPS, individual and support; PUK, University Hospital for Psychiatry Zurich; SMI, severe mental illness; ZhEPP, Zürcher Eingliederungs Pilot Projekt, ZInEP, Zürcher Impulsprogramms zur nachhaltigen Entwicklung der Psychiatrie.
